# *Cryptococcus gattii*, Florida, USA, 2011

**DOI:** 10.3201/eid1903.121399

**Published:** 2013-03

**Authors:** Rajesh Kunadharaju, Ulyee Choe, Julie R. Harris, Shawn R. Lockhart, John N. Greene

**Affiliations:** Author affiliations: University of South Florida Morsani College of Medicine, Tampa, Florida, USA (R. Kunadharaju, U. Choe, J.N. Greene);; Centers for Disease Control and Prevention, Atlanta, Georgia, USA (J.R. Harris, S.R. Lockhart); and Moffitt Cancer Center, Tampa (J.N. Greene)

**Keywords:** Fungi, Florida, Cryptococcus gattii, pneumonia, meningitis, septic arthritis, osteomyelitis, yeast, mycosis

**To the Editor:** Cryptococcosis is a systemic mycosis most commonly caused by 2 species of encapsulated yeast: *Cryptococcus neoformans* and *C. gattii*. *C. gattii* is a globally emerging pathogen. In the United States, an outbreak of *C. gattii* infection caused by molecular type VGII has been ongoing since 2004, primarily in the Pacific Northwest (*1*). In addition, sporadic cases caused by molecular types VGI and VGIII have been reported in other areas, including North Carolina, Rhode Island, New Mexico, Michigan, Georgia, and Montana (*2*). We report a case of disseminated *C. gattii* VGIIb infection in the United States outside of the Pacific Northwest in an otherwise healthy Florida native who had no known travel to *C. gattii*–endemic areas.

In May 2011, a 50-year-old man sought care for 6 months of progressive pain, swelling, and deformity of the left thigh and stiffness of his left knee. His only recent trauma was a minor left lower extremity injury 2 years earlier when a horse rolled on him. However, he had no fracture, and the injury eventually healed without medical care. He also reported occasional productive coughing and smoking 1 pack of cigarettes per day for 30 years. The patient was born and raised in Pasco County, Florida, and had not traveled outside of Florida in 20 years. He reported working on a dairy farm and having regular exposure to horses and pigs. Imaging showed a possible fracture of his left femur at the same site as the horse-related injury 2 years earlier. Computed tomographic scan of the chest demonstrated mediastinal lymphadenopathy and multiple pulmonary nodules bilaterally.

The man underwent open biopsy and fixation of the left femur fracture. Arthrocentesis was performed on his left knee. The bone and joint fluid were full of India ink–positive encapsulated budding yeast. The serum cryptococcal antigen was 1:4,096 (reference value, negative). An HIV antibody test result was negative, and CD4 count was 800 cells/mL (reference 500–2,600 cells/mL). A lumbar puncture showed normal opening pressure, 27 leukocytes/mL (reference 0–5 cells/mL) (89% lymphocytes [reference 40%–80%]), protein 464 mg/dL (reference 15–45 mg/dL), and glucose 21 mg/dL (reference 40–70 mg/dL). The cerebrospinal fluid (CSF) cryptococcal antigen was 1:4,096 (reference, negative). Magnetic resonance imaging of the brain indicated mild enhancement of the lining of the lateral ventricles and mild dilatation. 

*C. gattii* was isolated from the femur wound (superficial and deep) and CSF. Phenotypic testing was performed at ARUP Laboratories (Salt Lake City, UT, USA). In addition, the isolate was identified by multilocus sequence typing analysis as *C. gattii* type VGIIb by the Centers for Disease Control and Prevention (Atlanta, GA, USA) (*3*) (Figure).

**Figure:**
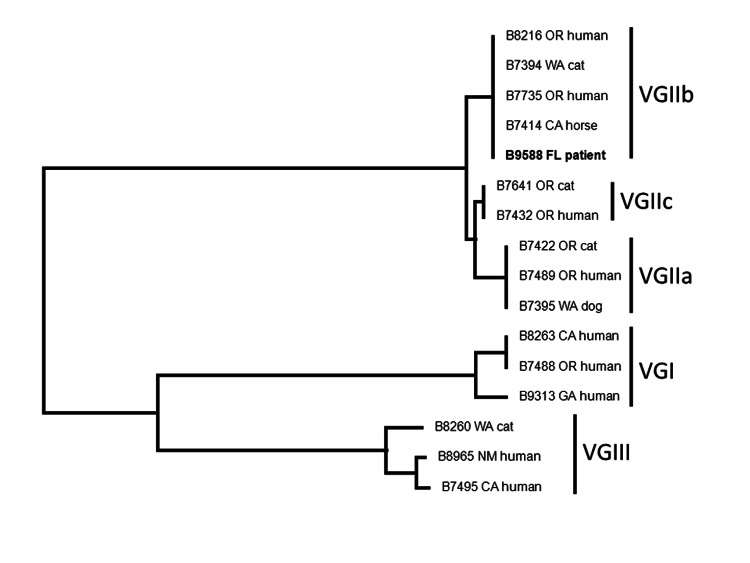
Neighbor-joining dendrogram of FL isolate (B9588, in **boldface**) with other US isolates showing that the FL isolate is identical to the VGIIb isolates from the US Pacific Northwest. The dendrogram was constructed by using multilocus sequence typing (*3*). FL, Florida; OR, Oregon; WA, Washington; CA, California; GA, Georgia; NM, New Mexico.

The patient was treated with liposomal amphotericin B and 5-flucytosine for 4 weeks for disseminated *C. gattii* infection with musculoskeletal, central nervous system, and pulmonary involvement. Repeat lumbar puncture revealed a normal opening pressure. CSF studies were not performed on this specimen. The patient gradually improved and was discharged on oral voriconazole (to be continued for 1 year) after 4 weeks of hospitalization. By July 2011, the patient was walking with crutches and had no symptoms other than persistent swelling and pain of his left leg. As of July 2012, he had fully recovered except for some residual pain and weakness in his left leg.

In addition to its newfound endemicity in the US Pacific Northwest, *C. gattii* is known to be endemic to Australia, Papua New Guinea, South and Southeast Asia, and some parts of Mexico and southern California (*4*). Its genetic diversity, the global distribution of isolates, and a broad range of hosts contribute to its success as a pathogen. *C. gattii* can be subdivided into at least 4 molecular types: VGI, VGII, VGIII, and VGIV (*5*). Most isolates identified from the Pacific Northwest outbreak are molecular type VGII, primarily comprising 3 distinct clonal subtype lineages: VGIIa, VGIIb, and VGIIc (*6*,*7*).

The case reported here involved *C. gattii* (VGIIb) outside the Pacific Northwest or other regions to which it is known to be endemic. Although the source of this patient’s infection remains unknown, his previous horse-related injury is intriguing as a possible source (*8*). All 4 isolates from horses in the Centers for Disease Control and Prevention’s collection are molecular type VGIIb (S.R. Lockhart, unpub. data). Other infections have been reported to seed the body and proliferate in areas of prior injury (*9*); this patient could have inhaled the cryptococcal yeast during exposure to horses, which then disseminated and seeded his prior injury site.

Clinically, infection caused by *C. gattii* outbreak strains (VGIIa/b/c) is characterized primarily by pulmonary complaints and pneumonia, with or without meningitis (*10*); other strains, such as VGI, occur as CNS disease (*10*). The patient reported here showed mainly musculoskeletal complaints, although involvement of the CNS and pulmonary systems was later found. Continued surveillance for *C. gattii* outside the Pacific Northwest will help shed more light on the spectrum of clinical manifestations. In the United States, *C. gattii* is likely to be seen increasingly outside the Pacific Northwest and other regions to which it is endemic.
